# Cardiovascular and comorbid risk factors for thrombosis in primary immune thrombocytopenia: a single-center retrospective study

**DOI:** 10.1016/j.rpth.2026.106940

**Published:** 2026-09-03

**Authors:** Ping Zhang, Yitong Xu, Mei Wang, Kun Zheng, Yue Lei, Yijia Chen, Ming Hou, Yu Hou

**Affiliations:** 1 Department of Hematology, Qilu Hospital of Shandong University, Shandong University, Jinan, China; 2 Cheeloo College of Medicine, Shandong University, Jinan, China; 3 State Key Laboratory for Innovation and Transformation of Luobing Theory, Jinan, China; 4 Shandong Key Laboratory of Hematological Diseases and Immune Microenvironment, Jinan, China

**Keywords:** arterial thrombosis, cardiovascular risk factor, comorbidity, primary immune thrombocytopenia (ITP), venous thrombosis

## Abstract

**Background:**

There is evidence that patients with immune thrombocytopenia (ITP) are at increased risk of thrombosis. The associations of clinical and comorbid factors with thrombosis in ITP remain unclear.

**Objectives:**

This study aimed to investigate the incidence rate and risk factors of arterial and venous thrombosis among patients with primary ITP.

**Methods:**

A retrospective cohort study was conducted, enrolling adult patients diagnosed with ITP at Qilu Hospital between January 1, 2014, and December 31, 2025. Thrombosis incidence was calculated using person-years of follow-up, and multivariable-adjusted Cox regression was used to identify independent risk factors. Sensitivity analyses were also conducted using Fine-Gray subdistribution hazard model regression, with death as a competing event.

**Results:**

Among 764 patients with ITP with 3540.8 person-years of follow-up, the incidence was 5.7 (95% CI, 4.9-6.5) per 100 person-years for arterial thrombosis and 2.1 (95% CI, 2.1 1.6-2.6) for venous thrombosis. The median age at thrombosis onset and the interval from ITP diagnosis to the index thrombotic event were 67.5 years and 2.4 years for arterial thrombosis and 62.0 years and 1.2 years for venous thrombosis, respectively. Recent 3-month exposure to all 3 ITP-targeted therapies increased the risk of both arterial and venous thrombosis.

**Conclusion:**

Advanced age, prior thromboembolism, comorbidities, recent exposure to ITP-targeted therapies, and cardiovascular risk factors were independent risk factors for thrombosis in ITP. Thromboembolism in ITP mostly occurred in the presence of multiple thrombotic risk factors. Continuous risk assessment and optimized management are essential throughout the clinical course of ITP.

## Essentials

• Patients with immune thrombocytopenia (ITP) have a higher risk of thromboembolism.
• A single-center retrospective cohort study was conducted to identify thrombotic risk factors in ITP.
• Advanced age, prior thromboembolism, and comorbidity confer high thrombosis risk factor.
• Continuous thrombotic risk assessment is essential throughout ITP clinical management.

## 1 Introduction

Immune thrombocytopenia (ITP) is an autoimmune hematological disorder characterized by a sustained platelet count below 100 × 10^9^/L, conferring an elevated bleeding risk [1]. Approximately 60% to 70% of adults with newly diagnosed primary ITP progress to chronic disease lasting over 12 months [2]. Beyond bleeding risk, patients with primary ITP also have a markedly elevated risk of arterial thrombosis (AT) and venous thrombosis (VT) relative to the general population [[3], [4], [5]]. The clinical management of ITP poses a therapeutic dilemma: thrombotic events substantially increase morbidity and mortality, whereas antithrombotic treatment may further heighten bleeding risk and adversely affect patient prognosis [6]. Given the high prevalence of thrombotic risk factors in patients with ITP, continuous risk assessment and mitigation throughout the disease course are essential [7].

Population-based cohort studies have demonstrated higher rates of AT and VT in patients with ITP compared with the general population [8,9]. Meanwhile, Chandan et al. [10] reported that individuals with ITP have a higher cardiovascular disease (CVD) burden, exhibiting an elevated risk of myocardial infarction and stroke compared with the general population. Likewise, a prospective cohort study assessing cardiovascular risk factors at ITP diagnosis demonstrated a high prevalence of such comorbidities in this patient population [11]. A Danish cohort study further demonstrated significantly elevated CVD-related mortality among patients with ITP [12]. In addition, Wang et al. [13] reported that a history of atherosclerotic disease and 3 or more conventional vascular risk factors were risk factors for AT in patients with ITP. These conditions correlate closely with increased AT incidence and cardiovascular mortality [13]. Therefore, causal studies remain warranted to clarify the risk factors for thrombosis in patients with ITP, including the risk of CVD.

Several individual and treatment-related risk factors contribute substantially to the occurrence of AT and VT in patients with ITP [14,15]. These risk factors include advanced age, exposure to thrombopoietin receptor agonists (TPO-RAs) [16], intravenous immunoglobulin (IVIg) [17], corticosteroid therapy [18], and splenectomy [19], as reported in multiple cohort studies. Splenectomy is a known risk factor for both VT [20,21] and AT [22] among patients with ITP. Thrombotic risk after splenectomy is nearly 3-fold higher than that of the general population [23]. These findings supported that ITP confers an inherent prothrombotic tendency modulated by treatment-specific factors, particularly recent exposure to ITP-targeted therapies.

To date, thrombotic risk factors in ITP remain incompletely elucidated and are generally categorized into individual-related, disease-specific, and treatment-related components [24]. Epidemiologically, acute cardiovascular embolism is highly prevalent in ITP, substantially compromising general health and quality of life [25]. Moreover, embolic events affecting vital organs including the heart and brain may severely impair quality of life and cause life-threatening complications [26].

Currently, clinical data on primary ITP complicated by thromboembolism in the Chinese population remain limited. Clarifying thrombotic risks and optimizing targeted prevention strategies hold important clinical implications. Accordingly, this study aimed to investigate the incidence, clinical characteristics, and risk factors of AT and VT in adult patients with primary ITP. In this Chinese cohort of patients with ITP, we evaluated the prevalence of AT and VT and explored their potential risk factors under comorbid conditions.

## 2 Methods

### 2.1 Identification of patients

Adult patients aged ≥18 years diagnosed with ITP were enrolled in this single-center retrospective study conducted at Qilu Hospital from January 1, 2014, to December 31, 2025. Patients with a documented diagnosis of ITP or idiopathic thrombocytopenic purpura were identified from the Qilu hospital digital medical record. Diagnostic criteria were based on the 2019 Updated International Consensus Report [27]. Patients with secondary ITP and those with incomplete clinical information were excluded, and all enrolled participants provided written informed consent. The study was approval from the institutional review board of Qilu Hospital, Shandong University (Approval No. KYLL-202307-009) and conducted in full compliance with the Declaration of Helsinki.

### 2.2 Outcome definition

Arterial thromboembolism was defined as a confirmed diagnosis of cerebrovascular accident, transient ischemic attack, ST-elevation myocardial infarction (STEMI), non-STEMI, acute myocardial ischemia, splenic infarction, bowel infarction, and peripheral AT. Venous thromboembolism (VTE) comprised confirmed cases of deep vein thrombosis, pulmonary embolism, cerebral venous sinus thrombosis, portal vein thrombosis, and splanchnic vein thrombosis. All confirmed arterial and venous thromboembolic episodes occurring during inpatient admission, ambulatory clinic visits, or postdischarge follow-up were included in our analysis.

### 2.3 Risk factors for thrombosis

The clinical characteristics of patients with ITP were collected during follow-up. Age and body mass index (BMI) were analyzed as continuous variables in the multivariable Cox regression analysis. Then age at initial ITP diagnosis was further stratified into 4 predefined strata (18-39 years, 40-59 years, 60-74 years, and ≥75 years) using cutoffs adopted in prior relevant literature [22,28]. BMI was calculated using each patient’s height and weight measured at hospital admission. The patients were classified into underweight (BMI <18.5 kg/m^2^), normal (BMI 18.5-23.9 kg/m^2^), overweight (BMI 24.0-27.9 kg/m^2^), and obese (BMI ≥28.0 kg/m^2^) groups according to the recommended BMI cutoffs for Chinese adults [29]. Hyperglycemia was defined as fasting plasma glucose ≥6.1 mmol/L, glucose-lowering medication use, or a documented diagnosis of diabetes mellitus. Glycemic control was defined as a glycated hemoglobin value <7.0%. In the absence of available glycated hemoglobin records, adequate glycemic control was determined by persistently stable fasting blood-glucose levels ranging from 4.4 to 7.0 mmol/L. Suboptimally controlled glycemia was defined as failure to achieve target glycemic parameters among patients receiving antidiabetic medication. Hyperlipidemia was defined as serum total cholesterol ≥5.2 mmol/L, triglycerides ≥1.7 mmol/L, or low-density lipoprotein cholesterol ≥3.4 mmol/L or drug treatment for lipid abnormality. Controlled dyslipidemia was defined as serum total cholesterol <5.2 mmol/L, triglycerides <1.7 mmol/L, and low-density lipoprotein cholesterol <3.4 mmol/L, regardless of whether patients received lipid-lowering medications. Suboptimally controlled dyslipidemia was assigned to participants undergoing lipid-lowering therapy who still met any threshold for hyperlipidemia.

Potential risk factors for VT were selected based on previous literature, including age at ITP diagnosis (categorized as 18-39 years, 40-59 years, 60-74 years, and ≥75 years); sex; prior VT history; cancer history within the preceding 5 years; chronic kidney disease; prior surgery within 3 months; pulmonary infection; hip, pelvic, or lower-extremity fractures; corticosteroid exposure; IVIg; TPO-RAs (eltrombopag and romiplostim administered); and splenectomy. Recent ITP treatment exposure was defined as receipt of corticosteroids, IVIg, or TPO-RAs within 3 months before incident thrombosis onset or administrative censoring.

Potential risk factors for AT included age at ITP diagnosis, sex, prior CVD (including previous AT and coronary artery disease), cancer history within the preceding 5 years, diabetes mellitus, chronic kidney disease, corticosteroid exposure, IVIg, TPO-RAs, and splenectomy. Cardiac risk factors were defined and assessed in accordance with the Framingham Heart Study criteria, encompassing smoking, obesity, hypertension, and prior acute myocardial infarction or cerebrovascular accident [30]. Hypertension was defined as systolic blood pressure (BP) ≥140 mm Hg and/or diastolic BP ≥90 mm Hg or use of antihypertensive medication [31]. Control of BP was defined as BP <140/90 mm Hg in subjects with medical treatment for hypertension. Suboptimally controlled BP was defined as BP ≥140 mm Hg and/or diastolic BP ≥90 mm Hg or the absence of antihypertensive medication.

### 2.4 Statistical analysis

Incidence rates (IRs) and IR ratios (IRRs) were estimated for the overall ITP cohort and further stratified by baseline risk factors. IRs were calculated according to person-years of follow-up and reported per 100 person-years with 95% CIs. Person-years at risk were accrued from the date of ITP diagnosis to the first occurrence of the study endpoint, including VT, AT, death, or loss to follow-up. Patients were censored at the time of the first endpoint event. Only the first episode of each thrombotic event occurring after ITP diagnosis was included in the analyses. Poisson regression models were applied to compute crude and adjusted IRRs (aIRRs). The median follow-up duration and platelet count were calculated and presented as median with IQR.

We first performed univariable Cox proportional hazards regression analysis for all potential risk factors and confounders. Variables with statistical significance (defined as *P* < .05) were subsequently incorporated into the multivariable Cox proportional hazards regression model to account for variable correlation. The adjusted hazard ratio (aHR) and corresponding 95% CIs were calculated to quantify the instantaneous relative hazard of AT/VT between exposed and reference groups. An aHR > 1 with a 95% CI entirely above 1 indicated a statistically significant increased thrombotic hazard. Sensitivity analyses were conducted using the Fine-Gray subdistribution hazard model, with death treated as a competing event. Stratified analyses by age subgroups (<60 years and ≥60 years) were additionally performed in both the multivariable Cox proportional hazards regression and the sensitivity analysis. Stratified Kaplan-Meier curves were used for the cumulative incidence of thrombotic events, with log-rank tests for between-group comparisons. All statistical analyses were performed using SPSS software, version 25.0 (IBM Corporation) and R software, version 4.1.0 (R Core Team). A 2-sided *P* value of <.05 was defined as statistically significant.

## 3 Results

### 3.1 Baseline characteristics of the patients with primary ITP

Of 764 enrolled adult patients with ITP, 58 were lost to follow-up and 32 died during follow-up, including 12 deaths from other causes, 12 from AT, and 8 from VT. The median follow-up from ITP diagnosis was 4.0 years (1.4-7.7 years), with a total observational follow-up of 3540.8 person-years. The median age at initial ITP diagnosis was 53.0 years (38.0-65.0 years); 60.9% of participants were female, whereas 61.8% (*n* = 473) were <60 years old and 38.1% (*n* = 291) were ≥60 years old. Among the overall cohort, 9.6% (*n* = 73) had a history of arterial infarction, and 3.8% (*n* = 29) had prior VT. CVD metabolic risk factors included hyperlipidemia (27.6%), hyperglycemia (20.2%), and hypertension (37.0%). With respect to treatment exposure, 84.2% of patients received corticosteroids, 47.8% received IVIg, and 56.5% were treated with TPO-RAs. Recent exposure to each agent within the 3-month window preceding thrombotic onset or administrative end of follow-up was observed in 169 patients (22.1%) for corticosteroids, 107 (14.0%) for IVIg, and 210 (27.5%) for TPO-RAs. Splenectomy was performed in 48 patients. Baseline characteristics and main clinical features of the study cohort are presented in Table 1.

**Table 1:** Characteristics of the patients with primary ITP.

Characteristics	Value
No. of patients	764
Ethnicity: Asian	764 (100)
Classification of ITP	
Newly diagnosed	66 (8.6)
Persistent	112 (14.6)
Chronic	586 (76.6)
Follow-up (y)	4.00 (1.4-7.7)
Sex	
Female	465 (60.9)
Male	299 (39.1)
Age (y)	53.0 (38.0-65.0)
Age group (y)	
18-59	473 (61.8)
60-74	237 (31.2)
≥75	54 (7.1)
BMI	24.8 (15.6-39.5)
CVD risk factors ≥3	193 (25.3)
History of arterial thrombosis	73 (9.5)
History of venous thrombosis	29 (3.8)
Hyperlipemia	211 (27.6)
Hyperglycemia	154 (20.2)
Hypertension	283 (37.0)
Chronic kidney disease	39 (5.1)
History of cancer	47 (6.2)
Previous surgery within 3 months	84 (11.0)
Corticosteroids	643 (84.2)
Recent corticosteroid treatment	169 (22.1)
IVIg	365 (47.8)
Recent IVIg treatment	107 (14.0)
TPO-RAs	432 (56.5)
Recent TPO-RA treatment	210 (27.5)
Splenectomy	48 (6.3)
Arterial thrombosis	202 (26.4)
Venous thrombosis	74 (9.7)
Platelet count at	54 (40-117)
Platelet count at VT	52.5 (30.8-73.3)
Death	32 (4.2)

Data are presented as *n* (%) and median (IQR).

Platelet count: median (IQR 25th-75th centile).

Recent treatment: recent exposure to ITP therapies within 3 months before incident thrombosis or end of follow-up.

AT, arterial thrombosis; BMI, body mass index; CVD, cardiovascular disease; ITP, immune thrombocytopenia; IVIg, intravenous immunoglobulin; TPO-RA, thrombopoietin receptor agonist; VT, venous thrombosis.

### 3.2 Incidence rate of thrombotic events

A total of 202 patients presented with incident AT and 74 with VT. The overall IR of AT among patients with primary ITP was 5.7 (95% CI, 4.9-6.5) per 100 person-years. Stratified by sex, the IR of AT was 4.7 (95% CI, 3.8-5.6) in female patients and higher in male patients, at 7.5 (95% CI, 6.0-9.1). The overall IR of VT was 2.1 (95% CI, 1.6-2.6), with no significant sex-based difference in VT risk. The median platelet count at the time of AT and VT was 54 × 10^9^/L and 52.5 × 10^9^/L, respectively (Table 1). The cumulative incidences of VT and AT in patients with ITP are shown in the Supplementary Figure.

### 3.3 Characteristics of patients with ITP with arterial thrombosis

Those who developed AT had a median age of 67.5 years (57.0-75.0 years), with a median interval of 2.4 years (0.68-6.0 years) from ITP diagnosis to the initial event (Table 2). Of all 202 AT events, 67.8% (*n* = 137) were cerebral infarction and 31.2% (*n* = 63) were myocardial infarction. Stratified by the Oxfordshire Community Stroke Project classification, lacunar infarction was identified in 15 patients (7.5%), total anterior circulation stroke in 9 (4.4%), partial anterior circulation stroke in 16 (7.8%), and posterior circulation stroke in 15 (7.5%); the remaining 69 cases (34.2%) presented with unclassified cerebral infarction. In addition, 13 patients (6.4%) experienced transient ischemic attack. In terms of cardiac adverse events, 20 patients (9.9%) had acute myocardial ischemia, 26 (12.9%) had unclassified myocardial infarction, 4 (1.9%) had non-STEMI, and 13 (6.5%) had STEMI. Peripheral limb infarction was documented in only 2 patients (1.0%). The detailed classification of all AT events is presented in Supplementary Table 1.

**Table 2:** Risk factors associated with arterial thrombosis (AT) in patients with ITP.

Variables	AT patients	Non-AT patients	Person-time (y)	IRs (95% CI, low-high)	Multiple adjusted RR^a^ (95% CI)
All, *n* (%)	202 (26.4)	562 (73.6)	3540.8	5.7 (4.9-6.5)	
Follow-up years, median (IQR)	2.4 (0.68-6.0)	4.7 (2.0-8.0)	*—*	*—*	*—*
BMI, mean ± SD	25.2 ± 3.6	24.6 ± 3.9	*—*	*—*	*—*
Sex, *n* (%)					
Male	97 (48.0)	202 (35.9)	1301.0	7.5 (6.0-9.1)	0.63 (0.37-1.1)
Female	105 (52.0)	360 (64.1)	2239.8	4.7 (3.8-5.7)	Reference
Age (y), median (IQR)	67.5 (57.0-75.0)	49.0 (35.0-61.0)	*—*	*—*	*—*
Age group (y), *n* (%)					
18-39	15 (7.4)	190 (33.8)	1052.0	1.4 (0.8-2.4)	Reference
40-59	59 (29.2)	209 (37.2)	1299.8	4.5 (3.5-5.9)	2.1 (1.2-3.8)
60-74	98 (48.5)	139 (24.7)	1000.3	9.8 (7.9-11.9)	3.4 (1.9-6.1)
≥75	30 (14.9)	24 (4.3)	188.7	15.9 (10.7-22.7)	3.8 (1.9-7.6)
Age at AT (y),median (IQR)	64.0 (54.0-71.3)	49.0 (35.0-61.0)	*—*	*—*	*—*
History of arterial thrombosis, *n* (%)	Yes 64 (31.2) No 139 (68.8)	Yes 9 (1.6) No 553 (98.4)	Yes 273.1 No 3267.7	23.1 (17.7-29.5) 4.3 (3.6-5.0)	2.5 (1.8-3.5)
Chronic kidney disease, *n* (%)	Yes 16 (7.9) No 186 (92.1)	Yes 23 (4.1) No 539 (95.9)	Yes 135.5 No 3405.3	11.8 (6.7-19.2) 5.5 (4.7-6.3)	1.9 (1.2-3.1)
Hyperlipidemia, *n* (%)	Yes 82 (40.6) No 120 (23.0)	Yes 129 (59.4) No 433 (77.0)	Yes 807.7 No 2733.1	10.2 (8.1-12.6) 4.4 (3.6-5.3)	1.5 (1.1-2.0)
Lipid profiles control, *n* (%)	Yes 25 (12.4) No 57 (28.2)	Yes 41 (7.3) No 88 (15.7)	Yes 241.1 No 566.6	10.1 (7.6-13.0) 10.4 (6.7-15.3)	1.1 (0.75-1.8) 1.8 (1.3-2.5)
Hyperglycemia, *n* (%)	Yes 79 (39.1) No 123 (60.9)	Yes 75 (13.3) No 487 (86.7)	Yes 630.7 No 2910.1	12.5 (9.9-15.6) 4.2 (3.5-5.0)	1.5 (1.1-2.0)
Glycemic control, *n* (%)	Yes 58 (28.7) No 21 (10.4)	Yes 64 (11.4) No 11 (2.0)	Yes 510.1 No 120.7	11.4 (8.6-14.7) 17.4 (10.8-26.6)	1.4 (0.9 -2.0) 1.8 (1.2-2.9)
Hypertension, *n* (%)	Yes 116 (57.4) No 86 (42.6)	Yes 167 (29.7) No 395 (70.3)	Yes 1246.6 No 2294.2	9.3 (7.7-11.2) 3.7 (3.0-4.6)	1.4 (1.1-1.9)
Controlled blood pressure, *n* (%)	Yes 21 (10.4) No 95 (47.0)	Yes 112 (19.9) No 55 (9.8)	Yes 638.8 No 607.8	3.3 (2.0-5.0) 15.6 (12.6-19.1)	0.65 (0.41-1.1) 1.9 (1.3-2.6)
Smoking, *n* (%)	Yes 93 (46.0) No 109 (54.0)	Yes 107 (19.0) No 455 (81.0)	Yes 816.5 No 2724.3	11.4 (9.2-14.0) 4.0 (3.3-4.8)	2.6 (1.5-4.4)
Obesity (BMI ≥ 28.0 kg/m^2^), *n* (%)	Yes 49 (24.3) No 153 (75.7)	Yes 103 (18.3) No 459 (81.7)	Yes 658.6 No 2882.2	7.4 (5.5-9.8) 5.3 (4.5-6.2)	1.1 (0.80-1.6)
Corticosteroids, *n* (%)	Yes 169 (83.7) No 33 (15.7)	Yes 474 (84.3) No 88 (15.7)	Yes 3063.0 No 477.8	5.5 (4.7-6.5) 6.9 (4.8-9.7)	0.81 (0.55-1.2)
Recent corticosteroids, *n* (%)	Yes 69 (34.2) No 133 (65.8)	Yes 100 (17.8) No 462 (82.2)	Yes 177.7 No 3363.7	38.9 (30.3-49.3) 4.0 (3.3-4.7)	5.2 (3.1-8.7)
IVIg, *n* (%)	Yes 99 (49.0) No 103 (51.0)	Yes 266 (47.3) No 296 (52.7)	Yes 1683.6 No 1857.2	5.9 (4.8-7.2) 5.5 (4.5-6.8)	1.1 (0.78-1.4)
Recent IVIg, *n* (%)	Yes 41 (20.3) No 161 (79.7)	Yes 66 (11.7) No 496 (88.3)	Yes 125.5 No 3415.4	32.7 (23.5-44.3) 4.7 (4.0-5.5)	1.8 (1.1-3.1)
TPO-RAs, *n* (%)	Yes 105 (52.0) No 97 (48.0)	Yes 327 (58.2) No 235 (41.8)	Yes 1857.8 No 1683.1	5.7 (4.6-6.8) 5.8 (4.7-7.0)	0.84 (0.63-1.1)
Recent TPO-RAs, *n* (%)	Yes 59 (29.2) No 143 (70.8)	Yes 151 (26.9) No 411 (73.1)	Yes 423.7 No 3117.2	13.9 (10.6-18.0) 4.6 (3.9-5.5)	1.1 (0.75-1.7)
Splenectomy, *n* (%)	Yes 20 (9.9) No 182 (90.1)	Yes 28 (5.0) No 534 (95.0)	Yes 275.67 No 3265.15	7.3 (4.1-10.4) 5.6 (4.8-6.4)	1.3 (0.85-1.8)
Platelet count, median (IQR)	30 (11-55)	18 (6-38)	*—*	*—*	*—*
<20, *n* (%)	75 (37.1)	294 (52.3)	1565.0	4.8 (3.8-6.0)	Reference
20-49, *n* (%)	63 (31.2)	162 (28.2)	1058.4	6.0 (4.6-7.6)	0.99 (0.69-1.4)
50-99, *n* (%)	64 (31.7)	106 (18.9)	917.4	7.0 (5.4-8.9)	1.1 (0.76-1.5)

Platelet count: at initial ITP diagnosis, median (IQR 25th-75th centile).

Recent treatment: recent exposure to ITP therapies within 3 months before incident thrombosis or end of follow-up.

BMI, body mass index; ITP, immune thrombocytopenia; IR, incidence rate (per 100 person-years); IVIg, intravenous immunoglobulin; RR, risk ratio; TPO-RA, thrombopoietin receptor agonist.

a Adjusted for age, sex, smoking, BMI, history of arterial thrombosis, chronic kidney disease, hyperlipidemia, hyperglycemia, and hypertension.

### 3.4 Incidence rates of arterial thrombosis by age, sex, and CVD risk factors

Stratified analyses revealed a stepwise elevation in the risk of AT with advancing age. Relative to patients aged 18-39 years, the IR of AT was 3-fold higher in those aged 40-59 years, 6-fold higher in patients aged 60-74 years, and highest in individuals aged ≥75 years. Compared with patients without prior AT, individuals with a history of AT exhibited a significantly elevated risk of recurrent AT (aIRR = 2.6, 95% CI, 1.8-3.6), representing an approximate 1.6-fold relative increase in thrombotic risk. Baseline chronic comorbidities were associated with elevated AT incidence. The IR was 11.8 (95% CI, 6.0-17.6) for patients with chronic kidney disease vs 5.5 (95% CI, 4.7-6.3) in those without, with an aIRR of 1.9 (95% CI, 1.1-3.1). Likewise, hypertensive patients had an IR of 9.3 (95% CI, 7.6-11.0) compared with 3.8 (95% CI, 3.0-4.5) in normotensive patients, with an aIRR of 1.4 (95% CI, 1.1-1.9). We further evaluated the associations of hyperglycemia and hyperlipidemia with incident AT risk. Patients with hyperlipidemia demonstrated a significantly higher thrombotic risk relative to normolipidemic individuals, with an aIRR of 1.5 (95% CI, 1.1-2.0) (Table 2). Similarly, individuals with hyperglycemia showed a significantly elevated risk, with an aIRR of 1.5 (95% CI, 1.1-2.0).

Majority of arterial thrombotic events were attributable to uncontrolled CVD risk factors among patients with ITP. A total of 95 (47.0%) patients had uncontrolled hypertension. The crude IRs of AT were 15.6 (95% CI, 12.6-19.1) and 3.3 (95% CI, 2.0-5.0) per 100 person-years for participants with suboptimally controlled and well-controlled BP, respectively. After multivariable adjustment, the aIRR of AT was 1.9 (95% CI, 1.3-2.6) in patients with uncontrolled hypertension and 0.65 (95% CI, 0.41-1.1) in those with controlled hypertension, relative to participants without hypertension. Likewise, compared with individuals with normolipidemia, the aIRR of AT was 1.8 (95% CI, 1.3-2.5) in patients with suboptimally controlled hyperlipidemia and 1.1 (95% CI, 0.75-1.8) in those with controlled hyperlipidemia. Relative to patients with normal glycemia, the aIRR was 1.4 (95% CI, 0.99-2.0) for patients with well-controlled hyperglycemia and 1.8 (95% CI, 1.2-2.9) for those with suboptimally controlled hyperglycemia. Lifestyle factors including smoking were associated with an elevated risk of AT. The corresponding aIRR was 2.6 (95% CI, 1.5-4.4) for smoking compared with nonsmoking. Glucocorticoid exposure within the preceding 3 months was significantly associated with a higher incidence of incident AT, with an aIRR of 9.2 (95% CI, 3.3-25.9). IVIg exposure yielded an aIRR of 1.8 (95% CI, 1.1-3.1), whereas TPO-RA therapy was not linked to a meaningful rise in thrombotic risk. A history of splenectomy was not significantly associated with incident AT risk, with an aIRR of 1.3 (95% CI, 0.88-1.9). The IRs of AT stratified by risk factors are summarized in Table 2.

Stratified analyses of AT incidence were performed across platelet count subgroups. The crude AT IR was highest in patients with platelet counts ≥50 × 10^9^/L (7.0 per 100 person-years), relative to the remaining platelet strata. However, no statistically significant differences in aIRRs were observed when each platelet subgroup was compared with the severely thrombocytopenic group.

Figure 1 presents cumulative incidence curves for AT stratified by significant clinical risk factors. These risk factors include age strata, prior AT history, hyperlipidemia, hyperglycemia, smoking, and hypertension (Figure 1).

**Figure 1:**
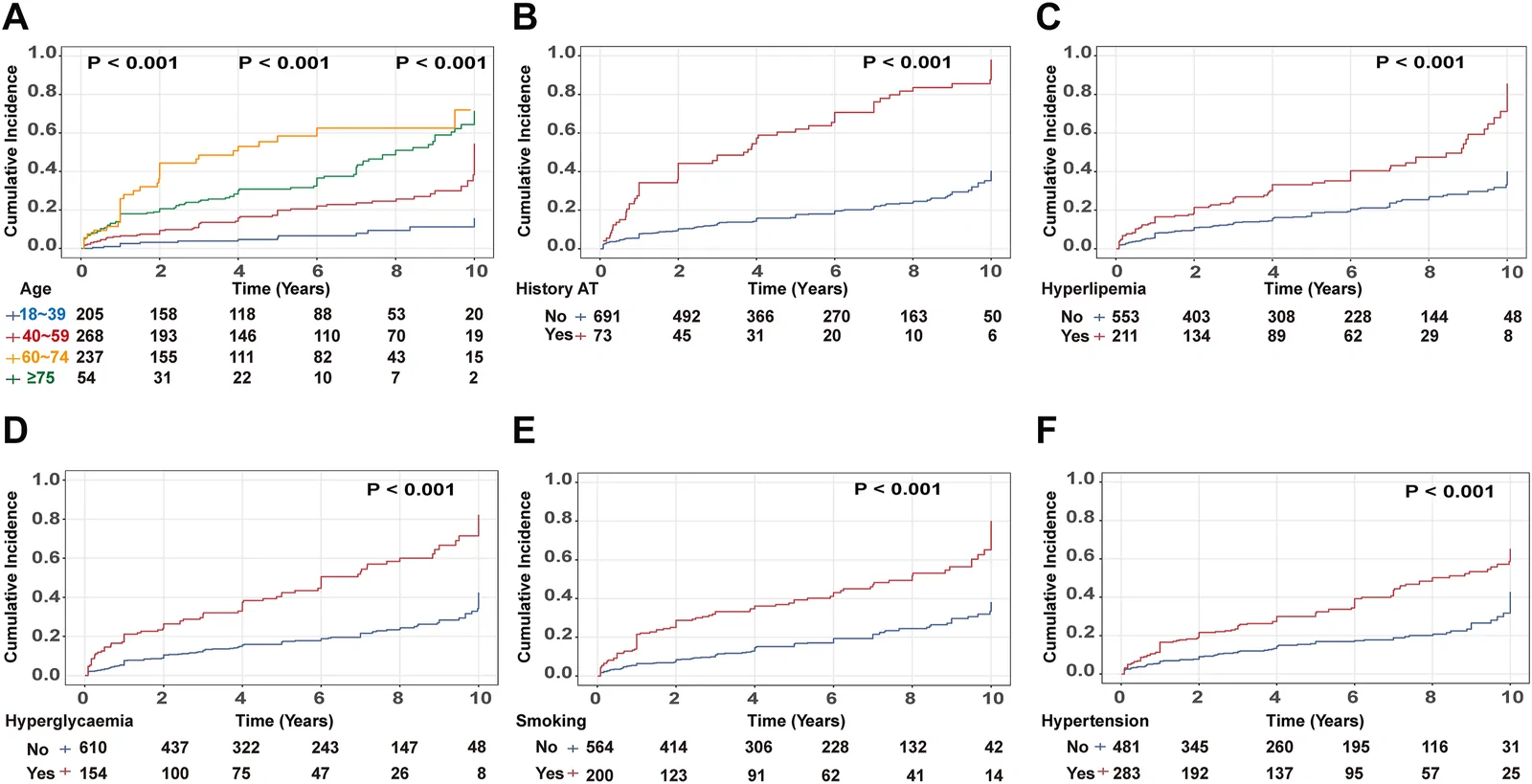
Cumulative incidence of arterial thrombosis (AT) in patients with immune thrombocytopenia stratified by risk factors. (A) Cumulative incidence of AT stratified by age group (*P*_(40-59 vs <40)_ < .001, *P*_(60-74 vs <40)_*<* .001, *P*_(≥75 vs <40)_ < .001). (B) Cumulative incidence of AT stratified by prior AT history (*P* < .001). (C) Cumulative incidence of AT in patients with and without hyperlipidemia *P* < .001). (D) Cumulative incidence of AT in patients with and without hyperglycemia (*P* < .001). (E) Cumulative incidence of AT in patients with and without smoking history (*P* < .001). (F) Cumulative incidence of AT in patients with and without hypertension (*P* < .001).

### 3.5 Baseline risk factors for arterial thrombotic events

Univariable analysis showed that age at ITP diagnosis, obesity, prior AT history, chronic kidney disease, hyperlipidemia, hyperglycemia, smoking, and hypertension were significantly associated with an increased risk of AT. Multivariable Cox regression identified increasing age as an independent risk factor for AT in patients with ITP. To quantify the graded association between advancing age and incident AT, age was categorized into 4 ordered groups (18-39, 40-59, 60-74, and ≥75 years) and incorporated as a categorical covariate in the multivariable Cox regression model (Supplementary Table 2). Patients aged 18-39 years were defined as the reference category. Compared with the reference group, aHRs were 2.3 (95% CI, 1.3-4.2; *P* = .006) for patients aged 40-59 years, 3.8 (95% CI, 2.1-8.0; *P* < .001) for those aged 60-74 years, and 4.0 (95% CI, 2.0-8.0; *P <* .001) for individuals aged ≥75 years. Additional independent predictors of AT after multivariable adjustment included hyperglycemia (aHR = 1.5; 95% CI, 1.1-2.1; *P* = .007), current smoking (aHR = 2.8, 95% CI, 1.7-4.8; *P <* .001), hyperlipidemia (aHR = 1.50; 95% CI, 1.1-2.1; *P* = .01), hypertension (aHR = 1.4; 95% CI, 1.1-1.9; *P* = .02), and a prior history of AT (aHR = 2.5; 95% CI, 1.8-3.4; *P <* .001). Univariable and multivariable Cox regression results are presented in Table 3.

**Table 3:** Variables associated with arterial thrombosis in patients with primary immune thrombocytopenia.

Variables	Univariable Cox model		Multivariable Cox model	
	HR (95% CI, low-high)	*P* value	HR (95% CI, low-high)	*P* value
Female	Reference	Reference	Reference	Reference
Male	1.5 (1.2-2.0)	.003	0.56 (0.33-0.93)	0.03
Age (y)	1.05 (1.04-1.06)	<.001	1.03 (1.02-1.04)	<.001
BMI	1.03 (1.00-1.07)	0.048	1.006 (0.97-1.0)	0.75
History of arterial thrombosis	5.2 (3.9-7.1)	<.001	2.5 (1.8-3.4)	<.001
Chronic kidney disease	2.0 (1.20-3.34)	.008	1.6 (0.96-2.7)	.07
Hyperlipidemia	2.4 (1.8-3.2)	<.001	1.5 (1.1-2.1)	.01
Hyperglycemia	3.0 (2.3-4.0)	<.001	1.5 (1.1-2.1)	.007
Smoking	2.9 (2.2-3.8)	<.001	2.8 (1.7-4.8)	<.001
Hypertension	2.5 (1.9-3.3)	<.001	1.4 (1.1-1.9)	.02

Crude incidence and HR using the Cox model.

BMI, body mass index; HR, hazard ratio.

Each cardiovascular metabolic risk factor was further categorized into 3 strata: disease absence, treated well-controlled status, and suboptimally controlled status. After multivariable adjustment, participants with suboptimally controlled cardiometabolic risk factors had a significantly higher hazard of incident AT, including suboptimally controlled hyperlipidemia (aHR = 1.7; 95% CI, 1.2-2.35; *P* = .003), uncontrolled hypertension (aHR = 2.0; 95% CI, 1.5-2.7; *P* < .001), and suboptimally controlled glycemia (aHR = 1.5; 95% CI, 1.1-2.1; *P* = .01). By contrast, no statistically significant excess thrombotic risk was observed in patients receiving pharmacotherapy with well-regulated lipid, BP, or glycemic levels relative to individuals free of the respective cardiometabolic comorbidity.

Sensitivity analysis using the Fine-Gray subdistribution hazard model regression yielded consistent effect directions, indicating that competing risks did not substantially influence the primary findings (Supplementary Table 3). Age subgroup analyses further validated these independent associations (Supplementary Table 4).

Thrombotic events predominantly occurred within 3 months of initiation of ITP rescue therapies (corticosteroids, IVIg, and TPO-RAs). Recent corticosteroid and IVIg exposure within 3 months was independently associated with a substantially elevated hazard of incident AT. The corresponding adjusted HR were 6.1 (95% CI, 4.1-9.0; *P* < .0001) and 1.9 (95% CI, 1.2-3.0; *P* = .026), respectively (Supplementary Table 2). However, no persistently elevated risk was observed among patients with remote historical treatment exposure (Supplementary Table 2).

### 3.6 Characteristics of patients with ITP with venous thrombosis

The median age of patients developing VT was 62.0 years (49.0-72.0 years), and the median duration from ITP diagnosis to the first event was 1.2 years (0.38-4.8 years). Of all 74 VT events, pulmonary embolism accounted for 9.5% (*n* = 7) and thrombophlebitis for 4.1% (*n* = 3). Peripheral extremity VT was the most common subtype, comprising 44 events (59.5%). Other venous embolism occurred in 8.1% (*n* = 6), portal VT in 8.1% (*n* = 6), and cerebral venous sinus thrombosis in 10.8% (*n* = 8). The incidence of VT stratified by age, sex, and comorbidity status is shown in Table 4.

**Table 4:** Risk factors associated with venous (VT) thrombosis in patients with ITP.

Variables	VT patients	Non-VT patients	Person-time (y)	IRs (95% CI, low-high)	Multiple adjusted RR^a^ (95% CI)
Venous thrombosis, *n* (%)	74 (9.7%)	690 (90.2%)	3533.69	2.1 (1.6-2.6)	
Follow-up years, median (IQR)	1.2 (0.38-4.8)	4.4 (1.7-7.9)	*—*	*—*	*—*
Sex, *n* (%)					
Male	24 (32.4%)	275 (39.9%)	1296.4	1.9 (1.9-2.8)	1.1 (0.58 -2.0)
Female	50 (67.6%)	415 (60.1%)	2237.3	2.2 (1.6-2.9)	Reference
Age (y), median (IQR)	62.0 (49.0-72.0)	53.0 (37.0-65.0)	*—*	*—*	*—*
Age group (y), *n* (%)					
18-39	11 (14.9)	194 (28.1)	1052.5	1.1 (0.43-1.7)	Reference
40-59	28 (37.8)	240 (34.8)	1293.6	2.2 (1.4-3.1)	1.4 (0.74-2.8)
60-74	28 (37.8)	209 (30.3)	996	2.8 (1.9-4.1)	1.4 (0.65- 2.8)
≥75	7 (9.5)	47 (6.8)	191.6	3.7 (1.5-7.5)	2.2 (0.71-7.0)
History of venous thrombosis, *n* (%)	Yes 20 (27.0) No 54 (73.0)	Yes 9 (1.3) No 681 (98.7)	Yes 92.5 No 3441.2	21.6 (13.2-33.4) 1.6 (1.2-2.0)	3.9 (2.3-6.4)
Bedridden state, *n* (%)	Yes 48 (64.9) No 26 (35.1)	Yes 63 (9.1) No 627 (90.9)	Yes 396.9 No 3136.8	12.1 (8.9-15.5) 0.83 (0.51-1.2)	6.0 (3.0-11.9)
Chronic kidney disease, *n* (%)	Yes 11 (14.9) No 63 (85.1)	Yes 28 (4.1) No 662 (95.9)	Yes 150.2 No 3383.8	7.3 (3.7-13.1) 1.9 (1.4-2.4)	2.3 (1.2-4.6)
Hip, pelvic, or lower-extremity fractures, *n* (%)	Yes 10 (13.5) No 64 (86.5)	Yes 9 (1.3) No 681 (98.7)	Yes 36.7 No 3497.0	27.3 (13.1-44.2) 1.8 (1.4-2.3)	2.3 (1.1-5.2)
Pulmonary infection, *n* (%)	Yes 33 (44.6) No 41 (55.4)	Yes 101 (14.6) No 589 (85.4)	Yes 529.6 No 3004.1	6.2 (4.3-8.8) 1.4 (0.98-1.9)	3.0 (1.7-5.4)
Previous surgery within 3 months, *n* (%)	Yes 32 (43.2) No 42 (56.8)	Yes 52 (7.5) No 638 (92.5)	Yes 306.2 No 3227.5	10.5 (7.1-14.8) 1.3 (0.94-1.8)	2.4 (1.3-4.6)
Corticosteroids, *n* (%)	Yes 50 (67.6) No 24 (32.4)	Yes 593 (85.9) No 97 (14.1)	Yes 3060.0 No 473.7	1.6 (1.6-2.7) 5.1 (3.3-7.5)	0.47 (0.23-0.95)
Recent corticosteroids, *n* (%)	Yes 24 (32.4) No 50 (67.6)	Yes 145 (21.0) No 545 (79.0)	Yes 157.1 No 3376.6	15.3 (9.8-22.7) 1.5 (1.1-2.0)	4.6 (2.1-10.3)
IVIg, *n* (%)	Yes 45 (60.8) No 29 (39.2)	Yes 320 (46.4) No 370 (53.6)	Yes 1700.7 No 1833	2.6 (1.9-3.5) 1.6 (1.1-2.3)	1.3 (0.76-2.3)
Recent IVIg, *n* (%)	Yes 20 (27.0) No 54 (73.0)	Yes 87 (12.6) No 603 (87.4)	Yes 125.5 No 3408.2	15.9 (9.7-24.6) 1.6 (1.2-2.1)	3.8 (1.5-9.8)
TPO-RAs, *n* (%)	Yes 30 (40.50) No 44 (59.5)	Yes 398 (57.7) No 292 (42.3)	Yes 1845.6 No 1688.1	2.6 (1.9-3.5) 1.6 (1.1-2.3)	0.75 (0.434-1.3)
Recent TPO-RAs, *n* (%)	Yes 27 (36.5) No 47 (63.5)	Yes 183 (26.5) No 507 (73.5)	Yes 419.5 No 3114.2	6.4 (4.2-9.4) 1.5 (1.1-2.0)	1.1 (0.60-2.0)
Splenectomy, *n* (%)	Yes 14 (18.9) No 60 (81.1)	Yes 34 (4.9) No 657 (95.2)	Yes 266.8 No 3266.9	5.2 (2.9-8.8) 1.8 (1.4-2.4)	1.1 (0.56-2.2)
Platelet count, median (IQR)	40.5 (19-60)	19 (7-42)	*—*	*—*	*—*
<20, *n* (%)	19 (25.7)	350 (50.7)	1557.5	1.2 (0.73-1.9)	Reference
20-49, *n* (%)	22 (29.7)	203 (29.4)	1061.1	2.1 (1.3-3.1)	1.3 (0.66-2.6)
50-99, *n* (%)	33 (44.6)	137 (19.9)	915.1	3.6 (2.5-5.1)	1.9 (0.89-3.9)

Platelet count: at initial ITP diagnosis, median (IQR 25th-75th centile).

Recent treatment: recent exposure to ITP therapies within 3 months before incident thrombosis or end of follow-up.

BMI, body mass index; IR, incidence rate (per 100 person-years); ITP, immune thrombocytopenia; IVIg, intravenous immunoglobulin; RR, risk ratio; TPO-RA, thrombopoietin receptor agonist.

a Adjusted for age, sex, BMI, previous surgery within 3 months, history of venous thrombosis, pulmonary infection, bedridden state, chronic kidney disease, and splenectomy.

### 3.7 Incidence rates of venous thrombosis by age, sex, and type of comorbidity

The IR of VT rose progressively with advancing age. Relative to the reference group of patients aged 18-39 years, the crude IR of VT was approximately 2-fold higher in patients aged 40-59 and 60-74 years, and highest among those aged ≥75 years. Patients with prior VT had a nearly 13-fold elevated VT risk, with an aIRR of 3.9 (95% CI, 2.3-6.4), compared with those without VT history. Bedridden state was also an independent risk factor for incident VT, with an aIRR of 6.0 (95% CI, 3.0-11.9). Similarly, individuals with postoperative status showed an approximately 8-fold higher risk (aIRR = 2.4; 95% CI, 1.3-4.6) relative to nonpostoperative patients. Prior hip, pelvic, or lower-extremity fractures conferred significantly elevated VT risk with an aIRR of 2.3 (95% CI, 1.1-5.2). Compared with patients without these conditions, significantly higher IRs of VT were observed in those with chronic kidney disease and pulmonary infection (Figure 2). Patients with ITP with chronic kidney disease exhibited a higher incidence of incident VT compared with those without chronic kidney disease (aIRR = 2.3; 95% CI, 1.2-4.6). Similarly, pulmonary infection was associated with a 3-fold increased VT IR (aIRR = 3.0; 95% CI, 1.7-5.4) among affected patients with ITP relative to those without pulmonary infection. Regarding ITP-specific therapies, elevated VT risk was identified among patients with ITP who received corticosteroids, TPO-RAs, or IVIg within the preceding 3-month exposure window, with aIRRs of 4.6 (95% CI, 2.1-10.3), 3.8 (95% CI, 1.5-9.8), and 1.1 (95% CI, 0.60-2.0), respectively. By contrast, remote historical exposure to these treatments was not associated with a significant increase in VT risk. Splenectomy was not significantly associated with an increased risk of VT among patients with ITP (aIRR = 1.1; 95% CI, 0.56-2.2), compared with patients who did not undergo splenectomy (Table 4).

**Figure 2:**
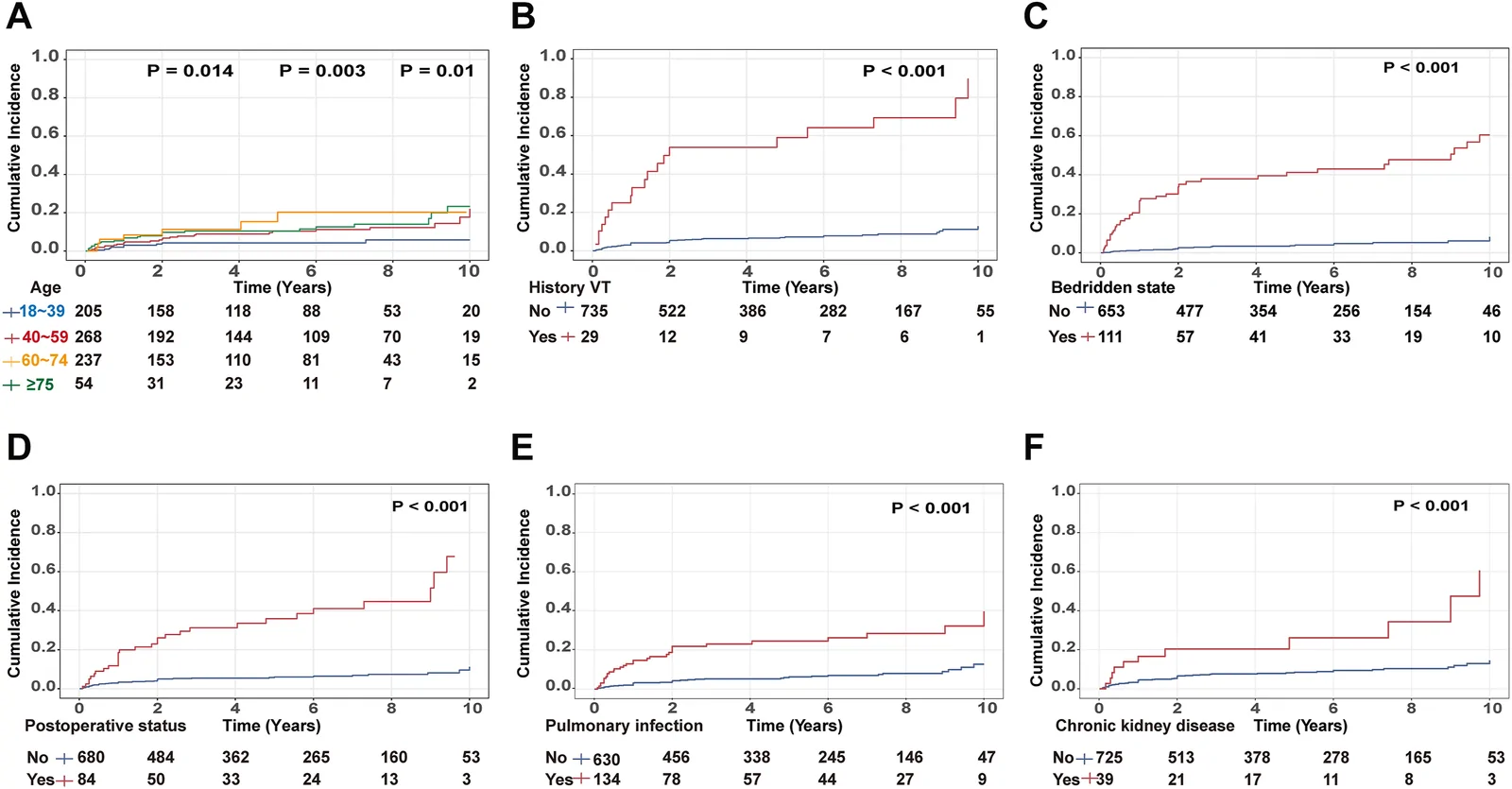
Cumulative incidence of venous thrombosis (VT) in patients with immune thrombocytopenia stratified by risk factors. (A) Cumulative incidence of VT stratified by age group (*P*_(40-59 vs <40)_ = .01, *P*_(60-74 vs <40)_ = .003, *P*_(≥75 vs <40)_ = .01). (B) Cumulative incidence of VT stratified by prior VT history (*P* < .001). (C) Cumulative incidence of VT in patients with and without bedridden state (*P* < .001). (D) Cumulative incidence of VT in patients with or without postoperative status (*P* < .001). (E) Cumulative incidence of VT in patients with and without pulmonary infection (*P* < .001). (F) Cumulative incidence of VT in patients with and without chronic kidney disease (*P* < .001).

We stratified VT incidence by platelet count subgroups. Although unadjusted VT IRs were numerically higher among patients with the mildly thrombocytopenic group (≥50 × 10^9^/L) compared with the reference group, none of the aIRRs indicated statistically significant intergroup differences.

Baseline comorbidities were associated with an elevated IR of VT among patients with ITP. Cumulative incidence curves for VT stratified by statistically significant risk factors, including age subgroups, prior VT history, bedridden status, postoperative state, pulmonary infection, and chronic kidney disease, are shown in Figure 2.

### 3.8 Baseline risk factors for venous thrombotic events

Univariable analysis showed that age at diagnosis, prior history of VT, postoperative status, pulmonary infection, bedridden state, renal disease, and splenectomy were significantly associated with an increased risk of VT (Table 5). Multivariable Cox regression analysis further identified age ≥75 years (aHR = 2.9; 95% CI, 1.1-7.9; *P* = .03), prior VT history (aHR = 3.4; 95% CI, 1.9-5.9; *P* < .001), bedridden state (aHR = 6.2; 95% CI, 3.4-11.3; *P* < .001), postoperative status (aHR = 1.8; 95% CI, 1.1-3.2; *P* < .001), pulmonary infection (aHR = 3.0; 95% CI, 1.9-4.9; *P* < .001), and chronic kidney disease (aHR = 2.1; 95% CI, 1.1-4.1; *P* = .03) as independent risk factors for VT in patients with ITP.

**Table 5:** Variables associated with venous thrombosis in patients with primary immune thrombocytopenia.

Variables	Univariable Cox model		Multivariable Cox model	
	HR (95% CI)	*P* value	HR (95% CI)	*P* value
Female	Reference	Reference	Reference	Reference
Male	0.78 (0.47-1.3)	0.32	*—*	*—*
BMI	0.97 (0.91-1.03)	0.32	*—*	*—*
Age (y)	1.03 (1.01-1.04)	.002	1.01 (0.99-1.03)	0.054
History of venous thrombosis	11.9 (7.0-20.1)	<.001	3.4 (1.9-5.9)	<.001
Previous surgery within 3 months	7.10 (4.4-11.3)	<.001	1.8 (1.1-3.2)	0.04
Pulmonary infection	4.6 (2.9-7.2)	<.001	3.0 (1.9-4.9)	<.001
Bedridden state	13.6 (8.4-22.1)	<.001	6.2 (3.4-11.3)	<.001
Chronic kidney disease	3.5 (1.8-6.7)	<.001	2.1 (1.1-4.1)	0.033
Splenectomy	2.6 (1.4-4.7)	.003	0.87 (0.43-1.8)	0.70

Crude and adjusted HR using the Cox model.

BMI, body mass index; HR, hazard ratio.

After full adjustment for confounders, short-term exposure to corticosteroids, IVIg, and TPO-RAs were all identified as independent risk factors for incident VT, whereas remote historical treatment exposure showed no significant thrombotic association (Supplementary Table 5). Corticosteroid administration within 3 months markedly elevated the hazard of VT (aHR = 7.3; 95% CI, 3.9-13.5; *P* < .001). VT hazard was increased 7.5-fold among patients receiving IVIg infusion within the prior 3 months (aHR = 7.5; 95% CI, 4.1-13.9; *P* < .001). Recent TPO-RA therapy within 3 months was associated with a 1.9-fold higher VT hazard (aHR = 1.9; 95% CI, 1.1-3.1; *P* = 0.02).

Univariable and multivariable Cox regression results are presented in Table 5. Fine-Gray subdistribution hazard regression revealed consistent effect estimates (Supplementary Table 6), and age-stratified subgroup analyses further confirmed these independent associations (Supplementary Table 7).

## 4 Discussion

In this retrospective cohort study of Chinese patients with primary ITP, we provided further evidence on the incidence of thrombotic events in this population. We observed IRs of AT and VT in adult patients with ITP. The IRs of both AT and VT in our study are mildly higher than that reported with findings reported from other countries. A meta-analysis of observational studies from both Europe and the United States reported the IR of AT to be in the range of 10.0 to 28.0 (per 1000 person-years) in patients with ITP. For VT, the meta-analysis reported an IR of 4.0-7.0 in patients with ITP [32]. The higher AT IR observed in the present study may be attributed to differences in population characteristics, including CVD risk factors and baseline comorbidity burden. Previous population-based studies are dependent on administrative health care databases, which limits the assessment of individual and metabolism-related factors that might account for the elevated thrombotic risk observed in patients with ITP.

Our analysis revealed that conventional cerebrovascular risks (hypertension, smoking, and prior AT), metabolic abnormalities (hyperglycemia, hyperlipidemia, and obesity), and chronic kidney disease were major contributors to AT. Previous cohort studies have demonstrated that patients with ITP frequently present with multiple cardiovascular risk factors including hypertension and diabetes [11,33] and have a significantly higher prevalence of renal failure than the general population [34]. Baseline characteristics of our ITP cohort were consistent with previously published reports. In our real-world, unselected ITP cohort, 73.95% of participants had at least 1 CVD risk factor, whereas 45.3% had 2 or more. Our findings are in line with data from the CARMEN clinical registry, which documented a high burden of CVD risk factors at ITP diagnosis, where 75.3% of patients had at least 1 cardiovascular risk factor [11]. Furthermore, prior studies have demonstrated that baseline CVD risk factors, malignancy [26], and renal disease [6] are each closely associated with an elevated risk of AT in patients with ITP [22], which is consistent with our results. Consistent with earlier evidence [19], our study highlights the clinical importance of recognizing baseline CVD risk factors for AT. Among cardiovascular risk factors at baseline, prior history of AT, hyperlipidemia, hyperglycemia, smoking, and hypertension were identified as independent predictors of AT in our cohort.

Suboptimal management of cardiovascular risk factors was highly prevalent among participants in our cohort, consistent with prior cohorts [35]. Specifically, 47.0% of patients with ITP who developed AT had suboptimally controlled BP, followed by suboptimal lipids control (28.2%) and poorly regulated glycemia (10.4%). Previous epidemiologic studies have confirmed that suboptimally controlled BP [36], substandard glycemic control [37,38], and untreated hyperlipidemia act as independent risk predictors for AT [39,40]. Consistent with prior cohorts, our present analysis identified suboptimally controlled traditional cardiovascular risk factors as an independent predictor of elevated arterial thrombotic risk in patients with ITP. Adequate management of these metabolic and vascular risk factors could substantially reduce the risk of AT.

Prior studies have consistently showed higher thrombotic rates in splenectomized individuals than nonsplenectomized counterparts [41,42]. This study verified that splenectomy increases the risk of both AT and VT. Prior VT, malignancy [43], and chronic kidney disease [6] are associated with an increased risk of VT [22,26]. Well-established conventional risk factors for VTE in the general population, including immobilization [44], a prior history of VTE, chronic kidney disease, and recent-onset pulmonary infection [14,19,45], also elevate the risk of VT among patients with ITP. After adjusting for multiple confounders in a multivariable regression model, we confirmed that advanced age, prior VT history, bedridden state, postoperative status, and comorbid conditions, such as pulmonary infection and chronic kidney disease, are significantly independent risk factors for VT.

Thrombotic risk increased progressively with advancing age [46]. We stratified patients with ITP by age to calculate the incidence density of thromboembolic events and compare thrombotic risks across different age subgroups [24]. Stratified analysis revealed a stepwise increase in thrombotic risk with advancing age in our study. Age ≥40 years was identified as an independent risk factor for incident AT, with excess thrombotic risk most prominent among patients aged ≥60 years. Correspondingly, age ≥75 years emerged as an independent predictor of VT within this ITP cohort. Accumulated evidence from prior cohorts demonstrates that patients with ITP aged ≥60 years carry a substantially elevated risk of thromboembolism [14,19,24,47]. Among patients with ITP, the median age at thrombotic onset was 67.5 years for AT and 62 years for VT. Our findings align with those from large population-based cohort studies conducted in Western populations [5,19,48].

Among thrombocytopenic adults, the aIRR for VT in the ≥50 × 10^9^/L subgroup was 2.0 (95% CI, 0.92-4.3); the corresponding aIRR for AT was 1.2 (95% CI, 0.83-1.8). There is no evidence for a direct relationship between disease severity and thrombotic risk. Approximately 42.0% of AT episodes and 49.5% incident VT occurred in patients with platelet counts <50 × 10^9^/L in our cohort, consistent with prior published data [43]. Patients with ITP face an elevated risk of bleeding, yet paradoxically they are also predisposed to thromboembolic events. In patients with ITP with multiple comorbidities, modification of ITP-specific therapy and thoughtful evaluation for anticoagulation therapy both are warranted [14,27].

Short-term exposure to ITP-specific therapies, including glucocorticoids, TPO-RAs and IVIg, within 3-month window before thrombotic onset was associated with elevated AT and VT risk in the present cohort. Consistently, previous real-world observational evidence demonstrated that a substantial proportion of thromboembolic events occurred in the early phase after TPO-RA initiation, within several weeks to 3-month follow-up [14,49,50]. Glucocorticoids could trigger transient hypercoagulability via endothelial injury and elevated procoagulant factors [18,22], thereby raising the risk of thromboembolic complications among patients with ITP [3,51]. Similarly, high-dose IVIg increases blood viscosity in the short term and further predisposes individuals to such thrombotic events [32,52,53].

The strengths of this study include the use of recent clinical data and a relatively large sample size, with comprehensive assessment of lifestyle-related risk factors (eg, smoking and BMI) and comorbidity-based risk profiles. The present study has several limitations. Owing to the retrospective design, timely and systematic assessment of disease activity and therapeutic outcomes could not be fully implemented, potentially introducing bias in the analysis of treatment-related thrombosis. One limitation of this study is the incomplete collection of serial dynamic platelet measurements around thrombotic events. For this reason, a definitive safe platelet count threshold could not be established from our data. Clinical antithrombotic decisions for patients with ITP with thrombosis should integrate bleeding history, thrombotic burden, and comorbidities.

Although thrombosis remains relatively uncommon in patients with ITP, it bears important clinical implications. Balitsky et al. [54] proposed a risk assessment score and highlighted the importance of comorbidities in ITP. Our observations suggest that comorbidity may increase the incidence of thrombosis in patients with ITP. This underscores the importance of routine evaluation and actively managing comorbid conditions in all diagnosed ITP individuals to mitigate subsequent thrombotic risk.
