# Supplementary material for: Cardiovascular and comorbid risk factors for thrombosis in primary immune thrombocytopenia: a single-center retrospective study
Source: Res Pract Thromb Haemost. 2026 Sep 3;10(6):106940. doi: 10.1016/j.rpth.2026.106940 (PMC13654523; doi:10.1016/j.rpth.2026.106940)
Supplement: Supplementary file 1 [file mmc1.docx]

**Supplemental Materials for**

**Cardiovascular and comorbid risk factors for thrombosis in primary immune thrombocytopenia：A single-center retrospective study**

**Authors:** Ping Zhang^1^, Yitong Xu^1^, Kun Zheng^1^, Mei Wang^1^,Yue Lei^4^, Yijia Chen^1^, Ming Hou^1,3^, and Yu Hou^1,2#^.

**1. Supplementary Figures**

Supplementary figure 1: Cumulative incidence curves of arterial thrombosis (A) and venous thrombosis (B) in patients with ITP.

**2. Supplementary Tables**

Supplementary Table 1. Categorization of arterial thrombosis subtypes in 202 ITP Patients.

Supplementary Table 2. Univariable and multivariable Cox-regression analyses were performed to assess independent risk-factors for arterial thrombosis among patients with ITP.

Supplementary Table 3. Variables associated with arterial thrombosis in patients with ITP using the Fine and Gray competing-risk model.

Supplementary Table 4. Variables associated with arterial thrombosis in patients with ITP using the Multivariable Cox model and Multivariable Fine and Gray model by age subgroups (patients <60 years and ≥60 years).

Supplementary Table 5. Univariable and multivariable Cox-regression analyses were performed to assess independent risk-factors for venous thrombosis among patients with ITP.

Supplementary Table 6. Variables associated with venous thrombosis in patients with primary ITP using the Fine and Gray competing-risk model.

Supplementary Table 7. Variables associated with venous thrombosis in patients with primary ITP using the Multivariable Cox model and Multivariable Fine and Gray model by age subgroups (patients <60 years and ≥60 years).

**Supplementary figure 1**

**Figure S1: Cumulative incidence curves of arterial thrombosis (A) and venous thrombosis (B) in patients with ITP**.

**Supplementary Table 1. Categorization of arterial thrombosis subtypes in 202 ITP Patients.**

| Number of patients | 202, 100% |
| --- | --- |
| Classification of ITP |  |
| Newly diagnosed | 46, 22.8% |
| Persistent | 23, 11.4% |
| Chronic | 133, 65.8% |
| Arterial thrombotic event types | - |
| Acute ischemic stroke (AIS） | - |
| TACS：Total Anterior Circulation Stroke | 9, 4.4% |
| PACS：Partial Anterior Circulation Stroke | 16, 7.8% |
| LACS：Lacunar Stroke | 15, 7.5% |
| POCS：Posterior Circulation Stroke | 15, 7.5% |
| Cerebral infarction (unspecified) | 69, 34.2% |
| Transient ischemic attack | 13, 6.4% |
| Acute myocardial ischemia | 20, 9.9% |
| STEMI | 13, 6.5% |
| Non-STEMI | 4, 1.9% |
| Myocardial infarction (unspecified) | 26, 12.9% |
| peripheral artery thrombosis (PAT) | 2, 1% |

Data are n , (%)

**Supplementary Table 2. Univariable and multivariable Cox-regression analyses were performed to assess independent risk-factors for arterial thrombosis among patients with ITP.**

| **Variables** | **Univariable Cox model** | | **Multivariable Cox model^a^** | |
| --- | --- | --- | --- | --- |
|  | **HR (95% CI)** | ***P value*** | **HR (95% CI)** | ***P value*** |
| Age group (years) |  |  |  |  |
| 18–39, n(%) | reference | reference | reference | reference |
| 40–59, n (%) | 3.5  (1.9–6.2) | *P<*0.001 | 2.3  (1.3–4.2) | 0.006 |
| 60–74, n (%) | 7.3  (4.2–12.7) | *P<*0.001 | 3.8  (2.1–6.8) | *P<*0.001 |
| ≥75, n (%) | 11.4  (6.0–21.5) | *P<*0.001 | 4.0  (2.0–8.0) | *P<*0.001 |
| BMI |  |  |  |  |
| underweight (BMI <18.5 kg/m^2^) | 0.50  (0.157–1.585) | 0.24 | 0.87  (0.27–2.79) | 0.82 |
| normal (BMI 18.5-23.9 kg/m^2^) | Reference | Reference | Reference | Reference |
| overweight (BMI 24.0-27.9 kg/m^2^) | 1.3  (0.91–1.7) | 0.17 | 1.1  (0.81–1.6) | 0.47 |
| obesity (BMI ≥28.0 kg/m^2^) | 1.498  (1.043–2.15) | 0.029 | 1.1  (0.78–1.7) | 0.49 |
| Normolipidaemic | reference | reference | reference | reference |
| Controlled lipid profiles | 2.4  (1.6–3.8) | *P<*0.001 | 1.3  （0.81–2.0） | 0.30 |
| Suboptimally-controlled lipid profiles | 2.3  (1.7 –3.2) | *P<*0.001 | 1.7  （1.2–2.4） | 0.003 |
| No hypertension | reference | reference | reference | reference |
| Controlled BP | 0.88  (0.55–1.4) | 0.61 | 0.66  (0.40–1.1) | 0.09 |
| Suboptimally-controlled BP | 3.9  (2.935–5.283) | *P<*0.001 | 2.0  (1.5–2.7) | *P<*0.001 |
| Normoglycaemia | reference | reference | reference | reference |
| controlled glycemic | 4.0  (2.5–6.4) | *P<*0.001 | 1.6  (0.93–2.58) | 0.093 |
| Suboptimally-controlled glycemic | 2.7  (2.0–3.7) | *P<*0.001 | 1.5  (1.10–2.12) | 0.011 |
| Corticosteroids | 0.92  (0.62–1.4) | 0.68 | 0.96  (0.63–1.5) | 0.86 |
| IVIG | 0.93  (0.71–1.2) | 0.63 | 0.87  (0.65–1.2) | 0.36 |
| TPO-RAs | 1.1  (0.81–1.4) | 0.62 | 0.94  (0.70–1.3) | 0.67 |
| Recent corticosteroids | 7.8  (5.7–10.8) | *P<*0.001 | 6.1  (4.1–9.0) | *P<*0.001 |
| Recent IVIg | 4.1  (2.9–5.8） | *P<*0.001 | 1.9  (1.2–3.0) | 0.026 |
| Recnt TPO-RAs | 2.3  (1.7–3.2) | *P<*0.001 | 1.2  (0.80–1.8) | 0.38 |

Recent treatment : recent exposure to ITP therapies within 3 months before incident thrombosis or end of follow-up; ^a^Adjusted for age, sex, smoking, BMI, History of arterial thrombosis,chronic kidney disease, hyperlipidemia, hyperglycaemia, hypertension for AT using multivariable Cox model.

**Supplementary Table 3. Variables associated with arterial thrombosis in patients with ITP using the Fine and Gray competing‑risk model.**

| **Variables** | **Number of events** | **Univariable**  **Fine and Gray model** | | **Multivariable**  **Fine and Gray model** | |
| --- | --- | --- | --- | --- | --- |
| Arterial thrombosis | 202, (26.4%) | **HR 95%CI** | **P value** | **HR 95%CI** | **P value** |
| Male | 97, (48.0%) | 1.5 (1.2–2.0) | 0.003 | 0.64 (0.36–1.2) | 0.14 |
| Age(years) | - | 1.05 (1.04–1.06) | *P*＜0.001 | 1.03 (1.02–1.04) | *P*＜0.001 |
| Age group (years) |  |  |  |  |  |
| 18–39, n(%) | 15, (7.4%) | reference | - | reference | - |
| 40–59, n (%) | 59, (29.2%) | 3.4 (1.9–5.9) | *P<*0.001 | 2.3 (1.3–4.0) | 0.005 |
| 60–74, n (%) | 98, (48.5%) | 6.9 (4.0–11.8) | *P<*0.001 | 3.6 (2.1–6.3) | *P<*0.001 |
| ≥75, n (%) | 30, (14.9%) | 9.9 (5.2–18.9) | *P<*0.001 | 3.2 (1.6–6.5) | 0.0013 |
| BMI | - | 1.03 (1.01–1.07) | 0.035 | 1.0 (0.97–1.04) | 0.77 |
| History of arterial thrombosis | 64, (31.2%) | 5.2 (4.0–6.9) | *P<*0.001 | 2.5 (1.8–3.4) | *P<*0.001 |
| Chronic kidney disease | 16, (7.9%) | 2.0 (1.7–3.4) | 0.011 | 1.6 (0.97–2.7) | 0.10 |
| Hyperlipemia | 82, (40.6%) | 2.4 (1.8–3.1) | *P<*0.001 | 1.5 (1.1–2.0) | 0.009 |
| Hyperglycaemia | 79, (39.1%) | 3.0 (2.3–4.0) | *P<*0.001 | 1.5 (1.1–2.1) | 0.013 |
| Smoking | 93,(46.0%) | 2.8 (2.1–3.6) | *P<*0.001 | 2.4 (1.3–4.2) | 0.004 |
| Hypertension | 116,(57.4%) | 2.4 (1.8–3.2) | *P<*0.001 | 1.5 (1.1–2.0) | 0.016 |

Data are n (%) or HR(95% CI low–high).

**Supplementary Table 4. Variables associated with arterial thrombosis in patients with ITP using the Multivariable Cox model and Multivariable Fine and Gray model by age subgroups (patients <60 years and ≥60 years).**

| **Variables** | **Patients < 60 years** | | | | **Patients ≥ 60 years** | | | |
| --- | --- | --- | --- | --- | --- | --- | --- | --- |
|  | **events, n** | **Multivariable Cox model HR (95%CI)** | **Multivariable**  **Fine and**  **Gray model^a^**  **HR (95%CI)** | **Fine and Gray model**  ***P* value** | **events,**  **n** | **Multivariable**  **Cox model**  **HR (95% CI)** | **Multivariable**  **Fine and Gray model**  **HR (95%CI)** | **Fine and Gray model**  ***P* value** |
| Arterial thrombosis | 74 | - | - |  | 128 | - | - | - |
| Male | 33 (44.6%) | 0.4  (0.15–0.96) | 0.36  (0.15–0.85) | 0.02 | 64 (50.0%) | 0.66  (0.33–1.33) | 0.73 (0.36–1.5) | 0.38 |
| Age | - | 1.04  (1.01–1.06) | 1.04  (1.0–1.1) | 0.004 | - | 1.02  (0.99–1.06) | 1.02  (0.99–1.05) | 0.29 |
| BMI | - | 1.02  (0.95-1.09) | 1.02  (0.97–1.1) | 0.48 | - | 1.0  (0.95-1.06) | 0.98  (0.94–1.04) | 0.55 |
| History of arterial thrombosis | 22 (29.7%) | 3.2  (1.8–5.8) | 4.0  (2.1–7.5) | *P<*0.001 | 41 (32.0%) | 2.3  (1.5–3.3) | 2.2  (1.5–3.3) | 0.004 |
| Chronic kidney disease | 7 (9.5%) | 2.2  (0.98–5.1) | 2.0  (0.82–4.8) | 0.11 | 9 (7.0%) | 1.2  (0.59–2.3) | 1.2 (0.64–2.3) | 0.60 |
| Hyperlipemia | 33 (44.6%) | 2.5  (1.5–4.2) | 2.6  (1.6–4.4) | 0.0002 | 49 (38.3%) | 1.1 (0.74–1.6) | 1.1 (0.76–1.5) | 0.67 |
| Hyperglycaemia | 16 (21.6%) | 1.2  (0.68–2.2) | 1.2  (0.62–2.2) | 0.63 | 63 (49.2%) | 1.9  (1.3–2.8) | 1.8  (1.3–2.6) | 0.0008 |
| Smoking | 31 (41.9%) | 4.2  (1.6–10.9) | 3.8  (1.6–9.4) | 0.003 | 62 (48.4%) | 2.4  (1.2–4.8) | 2.0 (0.97–4.0) | 0.06 |
| Hypertension | 30 (40.5%) | 1.6  (1.1–2.7) | 1.7 (1.07–2.9) | 0.04 | 86 (67.2%) | 1.3  (1.1–1.8) | 1.3 (0.90-1.9) | 0.16 |

Data are n (%) or HR(95%CI low–high).

**Supplementary Table 5. Univariable and multivariable Cox-regression analyses were performed to assess independent risk-factors for venous thrombosis among patients with ITP**

| **Variables** | **Univariable Cox model** | | **Multivariable Cox model^a^** | |
| --- | --- | --- | --- | --- |
|  | **HR (95% CI)** | ***P value*** | **HR (95% CI)** | ***P value*** |
| Age group (years) |  |  |  |  |
| 18–39, n(%) | reference | reference | reference | reference |
| 40–59, n (%) | 2.4 (1.1–4.9) | 0.02 | 1.7 (0.82–3.5) | 0.16 |
| 60–74, n (%) | 2.9 (1.4–5.9) | 0.004 | 1.6 (0.78–3.5) | 0.19 |
| ≥75, n (%) | 3.3 (1.3–8.7) | 0.015 | 2.9 (1.1–7.9) | 0.034 |
| BMI |  |  |  |  |
| underweight (BMI <18.5 kg/m^2^) | 0.38 (0.05–2.8) | 0.34 | 0.97 (0.13–7.3) | 0.98 |
| normal (BMI 18.5-23.9 kg/m^2^) | reference | reference | reference | reference |
| overweight (BMI 24.0-27.9 kg/m^2^) | 1.1 (0.67–1.8) | 0.68 | 1.1 (0.68–1.9) | 0.61 |
| obesity (BMI ≥28.0 kg/m^2^) | 0.63 (0.30–1.3) | 0.22 | 0.79 (0.37–1.7) | 0.57 |
| Corticosteroids | 0.34 (0.21–0.55) | P<0.001 | 0.89 (0.42–1.9) | 0.77 |
| IVIg | 1.6 (1.0–2.6) | 0.04 | 1.4 (0.86–2.4) | 0.17 |
| TPO-RAs | 0.69 (0.44–1.1) | 0.13 | 0.68 (0.42–1.1) | 0.12 |
| Recent Corticosteroids | 6.9 (3.8–12.4) | *P<*0.001 | 7.3 (3.9–13.5) | *P<*0.001 |
| Recent IVIg | 6.9 (3.9–12.2) | *P<*0.001 | 7.5 (4.1–13.9) | *P<*0.001 |
| Recent TPO-RAs | 2.8 (1.7–4.7) | *P<*0.001 | 1.9 (1.1–3.1) | 0.02 |
| Prior hip, pelvic or lower-extremity fractures | 11.3 (5.7–22.4) | *P<*0.001 | 1.9 (0.84–4.2) | 0.13 |

Recent treatment : recent exposure to ITP therapies within 3 months before incident thrombosis or end of follow-up; ^a^Adjusted for age, sex, BMI, previous surgery within 3 months, history of venous thrombosis, pulmonary infection, bedridden state,chronic kidney disease, and splenectomy for VT using multivariable Cox model.

**Supplementary Table 6. Variables associated with venous thrombosis in patients with primary ITP using the Fine and Gray competing-risk model.**

| **Variables** | **Number of events** | **Univariate**  **Fine and Gray model** | | **Multivariate* Fine and Gray model** | |
| --- | --- | --- | --- | --- | --- |
| venous thrombosis | 74, (9.7%) | **HR 95%CI** | ***P* value** | **HR 95%CI** | ***P* value** |
| Male | 24, (32.4%) | 0.78 (0.47–1.3) | 0.31 | 1.1 (0.63–1.9) | 0.75 |
| Age | - | 1.02 (1.0–1.04） | 0.003 | 1.01(1.0–1.03) | 0.11 |
| Age group (years) |  |  |  |  |  |
| 18–39, n(%) | 11, (14.9%) | reference | - | reference | - |
| 40–59, n (%) | 28, (37.8%) | 2.3 (1.1–4.6) | 0.019 | 1.7 (0.91–3.2) | 0.10 |
| 60–74, n (%) | 28, (37.8%) | 2.7 (1.3–5.4) | 0.005 | 1.5 (0.80–3.0) | 0.19 |
| ≥75, n (%) | 7, (9.5%) | 3.1 (1.2–8.1) | 0.02 | 2.7 (1.1–7.0) | 0.03 |
| History of venous thrombosis | 20, (27.0%) | 12.2 (7.3–20.3) | *P<*0.001 | 3.6 (2.0–6.3) | *P<*0.001 |
| Bedridden state | 48, (64.9%) | 13.0 (8.2–20.9) | *P<*0.001 | 5.9 (3.0–11.7) | *P<*0.001 |
| Previous surgery within 3 months | 32, (43.2%) | 7.07 (4.5–11.2) | *P<*0.001 | 3.7 (1.9–7.5) | 0.002 |
| Pulmonary infection | 33, (44.6%) | 4.4 (2.8–6.9) | *P<*0.001 | 2.9 (1.8–4.7) | 0.0006 |
| Chronic kidney disease | 11, (14.9%) | 3.4 (1.8–6.6) | *P<*0.001 | 2.4 (1.01–5.4) | 0.047 |
| Splenectomy | 14, (18.9%) | 2.6 (1.5–4.5) | 0.0007 | 1.2 (0.59–2.3) | 0.66 |

Data are n (%) or HR(95%CI low–high).

**Supplementary Table 7. Variables associated with venous thrombosis in patients with primary ITP using the Multivariable Cox model and Multivariable Fine and Gray model by age subgroups (patients <60 years and ≥60 years).**

| **Variables** | **patients < 60 years** | | | | **Patients ≥ 60 years** | | | |
| --- | --- | --- | --- | --- | --- | --- | --- | --- |
|  | **events,**  **n** | **Multivariable**  **Cox model**  **HR (95%CI)** | **MultivariableFine and**  **Gray model**  **HR (95%CI)** | **Fine and**  **Gray model**  ***P* value** | **events,**  **n** | **MultivariableCox model**  **HR (95%CI)** | **Multivariable**  **Fine and Gray model**  **HR (95%CI)** | **Fine and**  **Gray model**  ***P* value** |
| Venous thrombosis events | 39 | - | - |  | 35 | - | - | - |
| Male | 12 (30.8%) | 1.05  (0.50–2.3) | 1.1 (0.53–2.5) | 0.76 | 12 (34.3%) | 0.82  (0.38–1.78) | 0.95 (0.43–2.0) | 0.90 |
| Age | - | 1.02  (0.99–1.05) | 1.02  (0.99–1.04) | 0.17 | - | 0.97  (0.45–2.1) | 1.04  (0.99–1.1) | 0.13 |
| History of venous thrombosis | 11 (28.2%) | 3.0  (1.2–7.4) | 3.5  (1.4–8.4) | 0.006 | 9  (25.7%) | 3.8  (1.5–9.4) | 4.1 (1.5–11.2) | 0.006 |
| Bedridden state | 13 (33.3%) | 6.1  (2.5–14.5) | 5.7  (1.9–16.9) | 0.002 | 19  (54.3%) | 6.0  (2.4–14.7) | 5.30 (2.31–12.14) | 0.0001 |
| Previous surgery within 3 months | 14 (35.9%) | 4.3  (1.8–10.4) | 4.2  (1.5–11.7) | 0.006 | 18  (51.4%) | 4.2  (1.9–9.4) | 4.0 (1.8–9.0) | 0.001 |
| Pulmonary infection | 17 (43.6%) | 3.9  (1.8–8.6) | 3.4  (1.6–7.2) | 0.002 | 16  (45.7%) | 2.7  (1.3–5.6) | 2.6 (1.3–5.3) | 0.01 |
| Chronic kidney disease | 7 (17.9%) | 3.5  (1.7–10.1) | 4.3  (1.3–13.6) | 0.015 | 4  (11.4%) | 1.4  (0.43–4.5) | 1.5 (0.53–4.5) | 0.43 |
| Splenectomy | 6 (15.4%) | 1.3  (0.40–4.3) | 1.4  (0.42–4.4) | 0.61 | 8  (22.9%) | 0.98  (0.36–2.7) | 1.1 (0.44–2.6) | 0.90 |

Data are n (%) or HR(95% CI low–high).
